# ATG16L1 governs placental infection risk and preterm birth in mice and women

**DOI:** 10.1172/jci.insight.86654

**Published:** 2016-12-22

**Authors:** Bin Cao, Colin Macones, Indira U. Mysorekar

**Affiliations:** 1 Department of Obstetrics and Gynecology and; 2 Department of Pathology and Immunology, Washington University School of Medicine, St. Louis, Missouri, USA.

## Abstract

The placenta is a barrier against maternal-fetal transmission of pathogens. Placental infections can cause several adverse pregnancy outcomes, including preterm birth (PTB). Yet, we have limited knowledge regarding the mechanisms the placenta uses to control infections. Here, we show that autophagy, a cellular recycling pathway important for host defense against pathogens, and the autophagy gene Atg16L1 play a key role in placental defense and are negatively associated with PTB in pregnant women. First, we demonstrate that placentas from women who delivered preterm exhibit reduced autophagy activity and are associated with higher infection indicators. Second, we identify the cellular location of the autophagy activity as being in syncytial trophoblasts. Third, we demonstrate that higher levels of autophagy and ATG16L1 in human trophoblasts were associated with increased resistance to infection. Accordingly, loss of autophagy or ATG16L1 impaired trophoblast antibacterial defenses. Fourth, we show that *Atg16l1-*deficient mice gave birth prematurely upon an inflammatory stimulus and their placentas were significantly less able to withstand infection. Finally, global induction of autophagy in both mouse placentas and human trophoblasts increased infection resistance. Our study has significant implications for understanding the etiology of placental infections and prematurity and developing strategies to mitigate placental infection–induced PTB.

## Abstract 2

Autophagy is an important component of placental host defenses and susceptibility to preterm birth in mice and humans.

## Introduction

Preterm birth (PTB), a leading global cause of perinatal morbidity and mortality, is commonly associated with intrauterine and placental infections. Normally, the placenta serves as a formidable barrier to protect the fetus from maternal-fetal transmission of pathogens, such as *Listeria monocytogenes* and *Toxoplasma gondii* (1, 2). The placental cells that facilitate this protection are the fetal syncytiotrophoblasts (STBs) in the syncytium that covers the villous surface of the placenta and, being in direct contact with maternal blood, helps form the maternal-fetal blood barrier. STBs are derived from differentiation and fusion of highly proliferative cytotrophoblasts (CTBs), which stem from the trophectoderm (3). A third type of trophoblasts, extravillous trophoblasts (EVTs), extravasate from the villi, remodel the maternal spiral arteries, and invade the maternal interface to facilitate maternal blood flow to the growing fetus. We and others have shown that STBs are less susceptible to infection than CTBs and EVTs (4, 5), but the mechanism underlying this differential susceptibility is unknown.

An important part of the host immune response to microbial infection is the cellular recycling system autophagy. During autophagy, double-membrane vesicles, termed autophagosomes, form around cytoplasmic debris, organelles targeted for destruction, and pathogens and then deliver their contents to lysosomes for degradation (6–8). Upon autophagosome formation, microtubule-binding protein light chain 3 (LC3) converts from the soluble form LC3-I to the lipidated form LC3-II; thus, the level of LC3-II is an indicator of autophagic activity, or flux, in cells. LC3-II levels are higher in placentas from pregnancies complicated by preeclampsia (9) and intrauterine fetal growth restriction (10), suggesting that autophagy plays a role in placental function. Autophagy-related 16-like 1 (ATG16L1), a ubiquitin ligase critical for autophagosome closure, is a key player in regulating the autophagic response to pathogens (7). Additionally, a common polymorphism in *ATG16L1* (rs2241880, Thr300Ala) that impairs its autophagy function is associated with rapid labor progression in pregnant women (11). However, whether autophagic flux in general, and ATG16L1 in particular, contributes to placental susceptibility to infection and PTB is unknown.

Here, we demonstrate that decreased autophagy in human placentas is associated with early PTB and that autophagic activity is normally high in STBs and is a key mechanism driving the antibacterial defense mechanisms in the syncytium. Additionally, we show in mice that ATG16L1 is required to combat placental infection and that reduced expression of ATG16L1 leads to PTB and increased infection susceptibility in *atg16l1*-deficient placentas. Together, our findings provide a regulatory link among placental infection, autophagy, and PTB.

## Results

### Premature birth is associated with decreased autophagy and ATG16L1 expression in the placenta.

**Figure 1:**
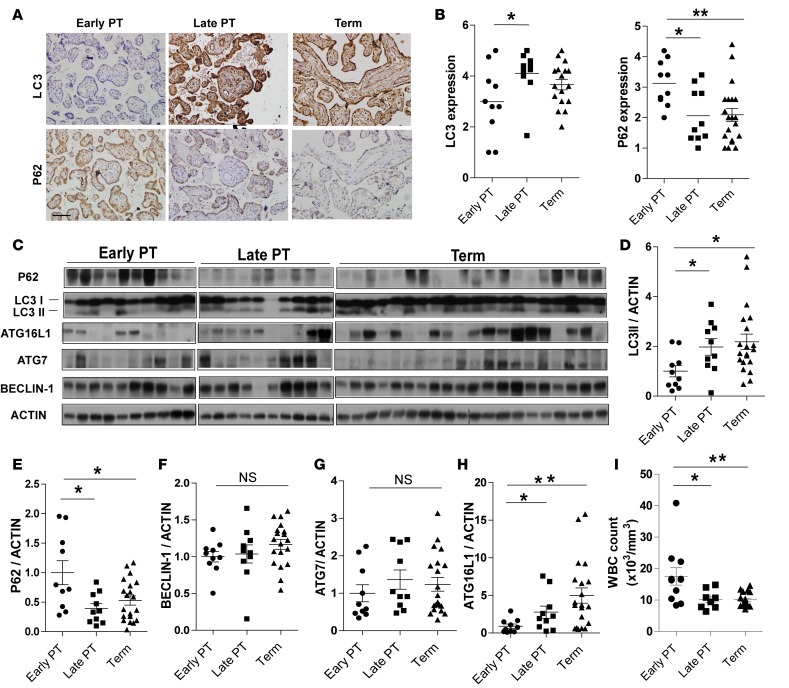
Autophagic activity is increased in preterm placentas. (**A**) Representative images of immunohistochemical staining of LC3 and P62 in placentas of early preterm (*n* = 10), late preterm (*n* = 10), and term deliveries (*n* = 20). Scale bar: 200μm. (**B**) Quantification of LC3 and P62 immunohistochemical staining. Staining images were examined and scored in a blinded fashion. Intensity of staining was scored from 1 (low) to 5 (high, P62) or 6 (high, LC3). (**C**) Western blot analysis of LC3-II, P62, ATG7, ATG16L1, BECLIN-1, and ACTIN from human placental samples from the indicated groups. (**D–H**) Quantification of indicated autophagy proteins normalized to ACTIN. (**I**) White blood cell (WBC) counts of patients in indicated groups. Data are expressed as mean ± SEM in **B**–**I**. *P < 0.05, **P < 0.01 using Kruskal-Wallis test with Dunnett’s post-test.

We collected placental samples from a cross-sectional cohort of 40 pregnancies from a single tertiary care hospital. Pregnant subjects were divided into three groups based on gestational age at delivery: early preterm (<32 weeks), late preterm (32–37 weeks), and term (>37 weeks) (Supplemental Table 1; supplemental material available online with this article; doi:10.1172/jci.insight.86654DS1). We examined the relationship between gestational age at birth and levels of autophagy as well as the association with white blood cell counts, a strong indicator of subclinical and clinical infections (12). To compare levels of autophagy among the three groups, we stained all placentas for LC3 and P62 (also known as SQSTM1), a linker protein that binds to ubiquitinated aggregates and targets them for degradation in the autolysosome (13). With increased autophagy, LC3-II levels increase and P62 levels decrease as P62-decorated organelles are degraded. Independent blinded quantification of immunohistochemical staining revealed that LC3 abundance was lower and P62 was higher in early preterm placentas than in late preterm and term placentas (Figure 1, A and B). Immunoblot analysis confirmed that P62 was higher and the LC3-II form of LC3 was lower in early preterm placentas than in late preterm and term placentas (Figure 1, C–E). The reduced level of autophagy in early preterm placentas was likely not simply due to low gestational age, as a study by Hung et al. showed that LC3-II and BECLIN-1 were expressed at all gestational ages (15 weeks to 40 weeks), and expression levels did not differ by gestational age (14). Thus, proper autophagy flux appears to be altered in early preterm placentas.

To further test this idea, we examined whether expression of key autophagy pathway genes or proteins differed in the preterm and term placentas. The canonical autophagy sequence involves assembly of autophagy-related proteins into complexes that are essential for steps of autophagosome formation. We found that protein levels of BECLIN-1 (involved in autophagy initiation) and ATG7 (involved in autophagosomal membrane elongation) did not significantly differ among early preterm, late preterm, and term placentas (Figure 1, C, F, and G). However, the average level of ATG16L1 was significantly lower in early and late preterm placentas than in term placentas (Figure 1H). Real-time quantitative PCR demonstrated that *ATG16L1* mRNA levels positively correlated with *LC3* mRNA levels (Supplemental Figure 1). Thus, ATG16L1 abundance correlates with autophagic activity in early preterm placentas.

From the clinical data on the patients, we observed that white blood cell counts, a strong indicator of subclinical and clinical intra-amniotic infections in PTB (12), were significantly higher in the women who delivered early preterm than in those who delivered late preterm or at term (Figure 1I and Supplemental Table 1). Notably, this was the case even though the percentage of women treated with antibiotics did not differ significantly in the three groups (Supplemental Table 1). Together, our findings suggest that a low level of autophagy, decreased expression of ATG16L1, and possibly subclinical infection in the placenta are associated with early PTB.

### Differential resistance in placental trophoblasts to infection is regulated by autophagy.

**Figure 2:**
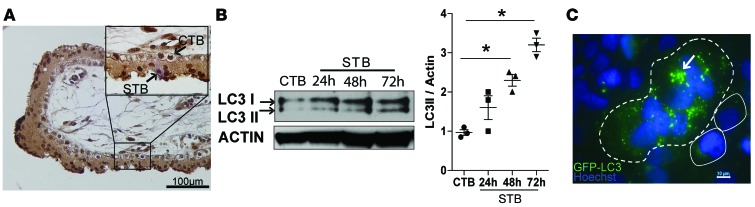
The baseline level of autophagy is higher in syncytialized than nonsyncytialized human trophoblasts. (**A**) Immunohistochemical staining of LC3 in a representative human term placental villus sample. Scale bar: 100 μm. Inset bar: 10 μM. (**B**) Western blot detection and quantification of LC3 levels in BeWo CTBs and STBs. (**C**) Immunofluorescent localization of the autophagy marker LC3 (green punctae) (arrow). Dashed line outlines STBs, solid lines outline CTBs. Blue, DAPI. Scale bar: 10 μm. Data are expressed as mean ± SEM.*P < 0.05 using Kruskal-Wallis test with Dunnett’s post-test.

Previously, we and others showed that trophoblast subtypes exhibit differential susceptibility to infection (4, 5). Given the link between autophagy and possible infection in preterm placentas, we reasoned that differential autophagy activation could underlie the differential susceptibility to infection in STBs and CTBs. To address this possibility, we performed immunocytochemistry of normal term human placenta samples and found that STBs expressed more LC3 than CTBs did (Figure 2A), suggesting that autophagy levels positively correlated with syncytialization. To further test this idea, we examined the levels of LC3-II and P62 in a well-established human choriocarcinoma cell line, BeWo, which is CTB-like and can be induced with forskolin to fuse to and form STBs (15). In BeWo cells, the level of LC3-II increased during the 72-hour time course of forskolin treatment (Figure 2B), confirming the positive correlation between autophagy activity and syncytialization. Finally, we found by immunofluorescence that BeWo STBs contained more LC3 punctae than CTBs, indicating increased LC3 lipidation and autophagy in STBs (Figure 2C). As an additional control to demonstrate that this phenomenon occurs in normal human trophoblasts, we isolated human primary mononucleated trophoblasts (PHTs) from normal full-term placentas. PHTs spontaneously form multinucleated STBs after 48 hours in culture. We found that P62 levels were lower in PHT-derived STBs than in CTBs (Supplemental Figure 2). To confirm that this finding indicated increased autophagic flux in STBs, we treated both cell types with bafilomycin, which inhibits autophagosome-lysosome fusion and thus stabilizes LC3-II. As anticipated, we found higher levels of LC3-II in STBs (Supplemental Figure 2). Together, our data suggest that autophagy activity increases during trophoblast syncytialization.

**Figure 3:**
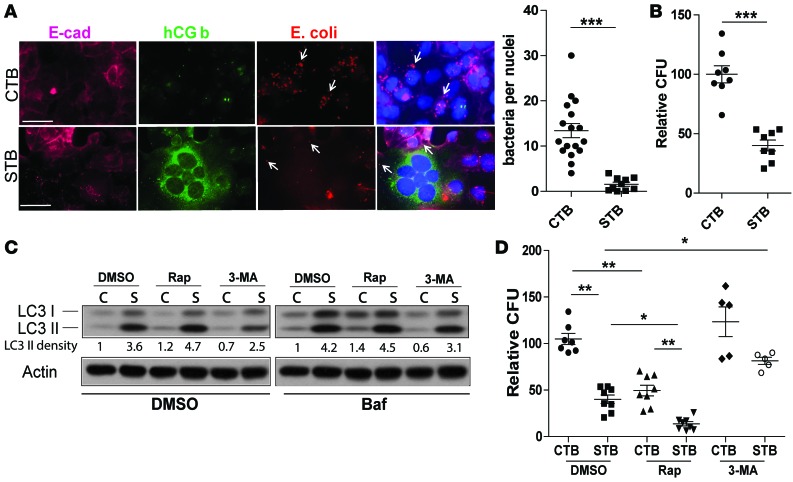
Autophagy contributes to STB resistance to bacterial colonization. (**A**) Immunofluorescence microscopy of CTBs (BeWo) and STBs (forskolin-treated BeWo) exposed to *E*. *coli* (red). STBs are marked by expression of human chorionic gonadotropin β subunit (hCG b, green), and cell membranes were marked by E-cadherin (magenta). The number of intracellular *E*. *coli* per trophoblast cell nucleus is shown. *n* = 200 nuclei/group. ***P < 0.001 by Mann-Whitney *U* test. (**B**) Number of intracellular *E*. *coli* determined by counting colony-forming units in STBs relative to CTBs (set at 100%). ***P < 0.001 by Mann-Whitney *U* test. (**C**) Western blot detection of LC3 in DMSO-, rapamycin- (Rap-), and 3-MA–treated CTBs and STBs. Bafilomycin (Baf) treatment indicates that enhanced LC3 activity is due to autophagic flux. (**D**) CFU analysis of intracellular bacteria number in CTBs upon rapamycin and 3-MA treatment. CFU counts were compared with DMSO-treated CTBs. Scale bar: 50 μm. Data are expressed as mean ± SEM. *P < 0.05, **P < 0.01 using Kruskal-Wallis test with Dunnett’s post-test.

Because autophagy is a vital part of the host response to infection in many cell types, we wanted to determine whether basal autophagy affected the trophoblast response to bacterial infection. We first infected CTBs and STBs with pathogenic *E*. *coli* and examined intracellular bacterial localization per cell by immunofluorescence staining (Figure 3A) and quantified bacterial load using colony-forming units (Figure 3B). We found that STBs contained significantly fewer intracellular bacteria than CTBs (Figure 3, A and B). Next, we treated the cells with an inducer, rapamycin, or inhibitor, 3-MA, of autophagy. As expected, rapamycin-treated CTBs and STBs both exhibited higher levels of LC3-II than DMSO-treated controls, and 3-MA–treated STBs had lower levels of LC3 II than control, DMSO-treated STBs (Figure 3C). This higher level of autophagy in STBs than in CTBs was maintained even upon bafilomycin treatment, indicating that it was not caused by impaired autolysosomal degradation (Figure 3C). Transmission electron microscopy (TEM) was performed and revealed that rapamycin treatment increased double-membraned autophagosome formation in CTBs, whereas 3-MA–treated CTBs had fewer fully developed autophagosome structures (Supplemental Figure 3).

We next examined bacterial load in these cells and found that rapamycin-treated CTBs and STBs both harbored lower intracellular bacterial burden than untreated cells, although CTBs still contained more bacteria than STBs (Figure 3D). Conversely, 3-MA treatment did not change the intracellular bacterial load in CTBs but did increase the bacterial load in STBs (Figure 3D). TEM analysis provides further evidence of direct interaction between bacteria and autophagosomes in trophoblasts (Supplemental Figure 3). Together, these data suggest that autophagy activity in placental cells is inversely correlated with susceptibility to bacterial infection and inhibition of autophagy impairs STB antibacterial defenses.

### Loss of ATG16L1 impaired antibacterial defenses in human and mouse placentas.

**Figure 4:**
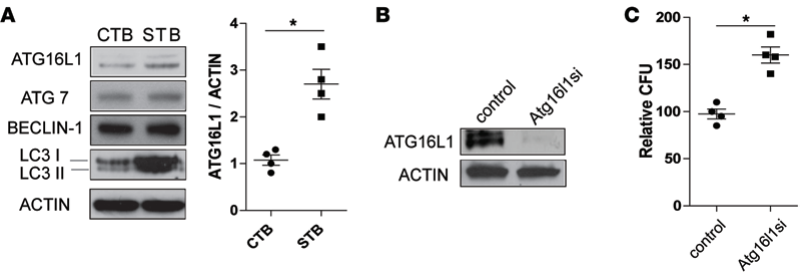
ATG16L1 in STBs regulates resistance to infection. (**A**) Western blot detection of autophagy proteins ATG16L1, ATG7, BECLIN-1 and LC3 in BeWo CTBs and STBs and quantification of ATG16L1 protein levels in CTBs and STBs. Mean values ± SEM of 3 independent experiments. (**B**) Western blot detection of ATG16L1 in STBs after exposure to control or *ATG16L1*-specific siRNA. (**C**) Quantification of bacterial load in STBs treated with control or *ATG16L1*-specific siRNA. The graph shows mean values ± SEM of 4 independent experiments. *P < 0.05, **P < 0.01 by Mann-Whitney *U* test.

We noted above that early preterm human placentas, which came from women with high white blood cell counts, had higher levels of ATG16L1 than term placentas. Moreover, examination of specific autophagy proteins revealed that levels of ATG16L1, but not ATG7 or BECLIN-1, were higher in STBs than CTBs (Figure 4A). To assess the requirement for ATG16L1 in governing STB resistance to infection, we used gene-specific siRNAs to knock down expression of *ATG16L1*. We found that loss of *ATG16L1* increased STB susceptibility to infection (Figure 4, B and C). Loss of *ATG16L1* in CTBs, however, did not alter CTB susceptibility to infection (data not shown). These data indicate that *ATG16L1* is an important component of STB resistance to bacterial infection.

**Figure 5:**
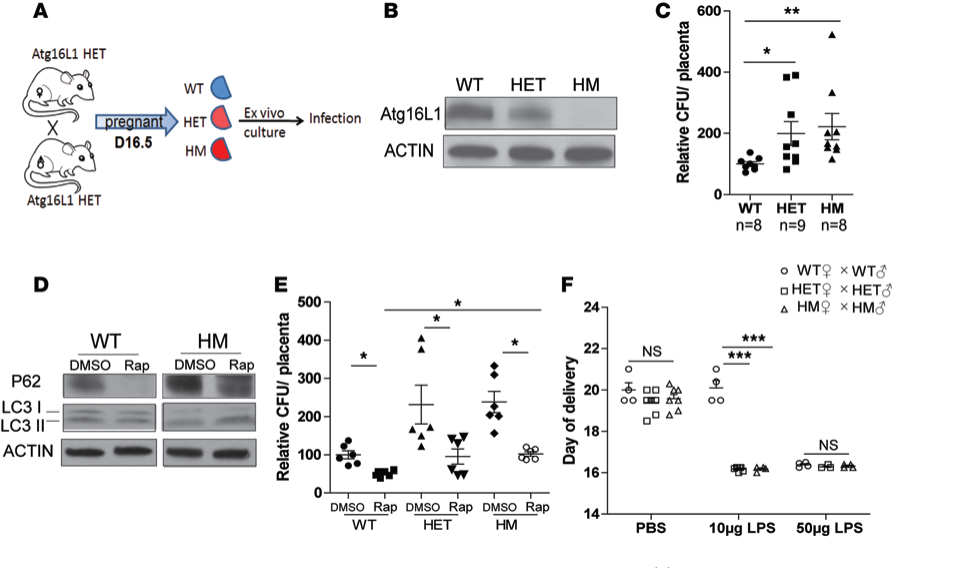
*Atg16l1-*deficient mice are susceptible to placental infection and LPS-induced preterm birth. (**A**) Schematic representation of mouse placental explant infections. (**B**) Western blot detection of ATG16L1 in placentas of the indicated genotype. (**C**) Quantification of intracellular *E*. *coli* load in placental explants of the indicated genotypes normalized to WT at 100%. *P < 0.05, **P < 0.01 by Kruskal-Wallis test with Dunnett’s post-test. (**D**) Western blot detection of LC3 and P62 in DMSO- and rapamycin-treated placental explants. (**E**) Quantification of intracellular *E*. *coli* load in DMSO- and rapamycin-treated WT, HET, and HM placental explants standardized to DMSO-treated WT. *P < 0.05 by Kruskal-Wallis test with Dunnett’s post-test. (**F**) Day of delivery in WT, HET, and HM mice injected with PBS or LPS on day 15.5 of pregnancy. Data are expressed as mean ± SEM. *P < 0.001 by 2-way ANOVA with a Bonferroni post-test.

To further assess the requirement for ATG16L1 in protecting the placental syncytium from infection, we used *Atg16L1*-hypomorphic (*Atg16L1^HM^*) mice, which display compromised autophagic activity in numerous tissues but no obvious phenotypic abnormalities in the absence of infection/injury (16). Mice heterozygous for *Atg16L1^HM^* were bred to generate pups and placentas with three genotypes: WT, heterozygous (HET), and homozygous (HM) (Figure 5A). Breeders gave birth to pups in normal Mendelian ratios, and placental and fetal weights were similar among the three genotypes (Supplemental Figure 4, A–C). Furthermore, the HM placentas exhibited normal histological structure, with similar thickness of the three functional layers — decidua, junctional zone, and labyrinth — as WT placentas (Supplemental Figure 4D). As expected, HET and HM placentas expressed less ATG16L1 protein than WT littermate placentas (Figure 5B). P62 levels were higher in both HM and HET placentas than in WT placentas, suggesting that even intermediate ATG16L1 deficiency can impair autophagy flux (Supplemental Figure 5). We conclude that ATG16L1 is necessary for autophagic activity in the placenta.

To elucidate whether ATG16L1 was also necessary for placental defense against bacterial pathogens, we developed an ex vivo mouse placental explant model that exhibits normal morphology and expression of the placental epithelial maker cytokeratin 7 (Supplemental Figure 6). We challenged explants of WT, HET, and HM littermate placentas with pathogenic *E*. *coli* and found that HM and HET placental explants both harbored significantly higher intracellular bacterial loads than WT explants (Figure 5C). We wondered whether higher sensitivity to bacterial infection in *Atg16L1*-deficient placentas was caused by compromised canonical autophagy or whether it could be rescued by induction of autophagic flux. Thus, we pretreated placental explants with rapamycin to globally induce autophagy, which we confirmed by examining P62 and LC3-II levels (Figure 5D). Upon rapamycin treatment, the numbers of intracellular bacteria in HM and HET placentas were reduced to levels similar to those in WT placentas (Figure 5E). Together, these findings suggest that ATG16L1 and the autophagy pathway in general contribute to protecting the placenta against bacterial infection.

### ATG16L1-deficient mice exhibit increased sensitivity to inflammation-induced PTB.

Our human placental studies described in Figure 1 suggested that premature placentas expressed lower levels of ATG16L1 than term placentas. Thus, we asked whether loss of *Atg16L1* would trigger spontaneous PTB in pregnant mouse dams. We crossed WT, HET, and HM females to males of the same respective genotypes and observed no differences in gestational length, indicating that *Atg16l1* deficiency alone did not trigger spontaneous PTB (Figure 5F). We wondered whether inflammation would synergize with loss of Atg16L1 to trigger PTB. Thus, we injected dams on day 15.5 of pregnancy with LPS, which is often used to induce inflammation in animal models of infection/inflammation-induced PTB (17). Whereas mice of all three genotypes gave birth within 24 hours after treatment with a high dose (50 μg) of LPS, only HET and HM mice delivered prematurely when given a lower dose of LPS (10 μg) (Figure 5F). Together, these findings suggest that loss or dysfunction in *Atg16L1* can lead to increased sensitivity to inflammation-triggered PTB.

## Discussion

Autophagy is a vital part of the host immune response that delivers intracellular pathogens to lysosomes, thereby eliminating them. However, the role of autophagy in protection from bacterial pathogens in the placenta was hitherto poorly understood. Here, we show that autophagy protects the placenta from bacterial infection, and levels of autophagy at least partly explain the differential susceptibility of trophoblast types to bacterial challenge. We conclude that, in addition to their dense and branched microvilli, lack of intercellular junctions, and dense physical actin network (18), STBs use autophagy to combat bacterial infections. The placenta is an immunoprivileged organ that protects the fetus from both maternal immune rejection and pathogen challenge. However, several of the pathogens that can infect the placenta, such as *B*. *abortus* (19), *C*. *burnetii* (20), and *L*. *monocytogenes* (21), can evade or subvert the autophagic cellular machinery to survive intracellularly in other cell types (22). Thus, high levels of autophagy in the placenta may explain why transplacental infections are rare even for autophagy-hijacking pathogens. Recent work from our laboratory has demonstrated that the emerging virus, Zika, can be successfully transmitted from mother to fetus in a transplacental manner via trophoblasts (23–25). It remains to be determined whether the Zika virus is able to cross the placenta by modifying autophagy.

Previous studies have suggested that autophagy is regulated during human pregnancy, especially during placental distress as a higher level of autophagy was observed in placentas from cesarean-sectioned mothers versus those that delivered vaginally (26). Higher placental autophagy activity has been associated with development of preeclampsia (9) and intrauterine growth restriction (10). Thus, although our studies suggest an important role for higher autophagic activity level in the placental syncytium in terms of host defense, there appears to be a complicated relationship between autophagy activity regulation and pregnancy outcomes in general. The precise mechanisms of spatial and temporal regulation by autophagy in pregnancy, placental infections, and noninfectious conditions needs be further investigated.

We have shown that loss of *Atg16L1* protects mice against infections (27) of the urinary tract and the intestine (28). However, *Atg16L1* deficiency in many other tissues leads to increased susceptibility to infection (29, 30). The data presented here show that the role of *Atg16L1* in the placenta is more similar to that in those other tissues, with loss of the gene leading to increased susceptibility to bacterial infection. Hence, this is further confirmation that the roles of ATG16L1 in bacterial infections are tissue dependent. Consistent with the idea that ATG16L1 plays an important role in placental function, women with the Thr300Ala (rs2241880) single nucleotide polymorphism in the *ATG16L1* gene had shorter induction times during labor than women with two other *ATG16L1* genotypes (11). This finding raises the possibility that regulation of *ATG16L1* affects labor timing in humans.

Even in the absence of infection, autophagy appears to be involved in birth timing in mice. For example, ovarian granulosa cell–specific Beclin-1 knockout mice exhibit impaired lipid droplet formation and significantly reduced progesterone synthesis, which leads to PTB (31). Additionally, treatment with rapamycin abolished preterm labor in an animal model of spontaneous PTB, namely P53 conditional knockout mice (32). Finally, dysregulation of autophagy has recently been shown in a mouse model of inflammation-induced preterm labor (33).

Infection and inflammation are thought to be the most important triggers for PTB. Our work provides the first evidence to our knowledge that autophagy activity and ATG16L1 expression are reduced in early preterm placentas. These placentas came from women with increased white blood cell counts, suggesting that loss or dysfunction of ATG16L1 can synergize with inflammation, and possibly infection, to cause PTB. This idea is consistent with our observation that Atg16L1^HM^ mice only delivered preterm when also treated with LPS and indicative of gene-environment interactions at play in determining the threshold for adverse birth outcomes. We suggest that because autophagy is a vital part of the host response to microbial infection and can directly eliminate intracellular pathogens by mediating their delivery to lysosomes, infection is a driving factor for high autophagic activity in the placenta. Thus, we must keep in mind that dysfunction or modulation of autophagy in response to a pathogen may play a role in PTB that is equal in importance to infection itself.

Trophoblasts express pattern recognition receptors such as Toll-like receptors (TLRs) that can recognize extrinsic microbial factors. TLR4 is the primary detector of LPS on Gram-negative bacteria. Trophoblasts also express intracellular receptors called Nod-like receptors (NLRs) that recognize bacterial products in the cytosol, and NLRs may serve as components of inflammasomes, which are key signaling complexes that detect pathogenic microorganisms and activate the highly proinflammatory cytokine IL-1β (34). Studies from our group and others have revealed that autophagy can dampen activation of the innate immune response to infection as well as activation of inflammasomes, in particular NLRP3 (6, 7, 35). Additionally, a recent study showed that exposing CTBs from first trimester placental samples to bacterial LPS upregulated NLRP3 expression and induced IL-1 β secretion, suggesting that the NLRP3 inflammasome is important in placental innate immune responses (36). Thus, the complex process of labor and birth may represent a combinatorial confluence of genetic and environmental causes. This idea has profound implications for how we view the relationship between genetic heterogeneity and PTB in humans and suggests that studies examining associations between prematurity and infections should also consider genetic variation. Further defining the roles of autophagy and key autophagy genes in the placenta will have significant implications for understanding the etiology of placental infections and developing strategies to mitigate placental infection–induced PTB.

## Methods

### Human samples.

Placental specimens were collected at Barnes-Jewish Hospital and processed through the Women and Infants’ Health Specimen Consortium. None of the PTB patients showed clinical or pathological features of other maternal or placental complications. Samples (5–8 mm) from the basal plate, chorionic villi, and amniotic membranes were obtained from placentas immediately after delivery. Tissues were processed as previously described (37).

### Mouse husbandry.

*Atg16l1^HM^* mice were provided by Herbert “Skip” Virgin (Washington University, St. Louis, Missouri, USA) (16). All mice were on a C57BL6 background and housed under a 12-hour-light/12-hour dark cycle in a clean mouse breeding facility. For mating, 12- to 15-week-old male and nulliparous female mice were cohoused from 5:00 pm to 8:00 am, and day 1 of pregnancy was defined on first observation of a vaginal plug. Mouse placenta samples were collected at day 16.5 and processed as described below.

### Analysis of birth timing.

Mice received intraperitoneal injection of saline or LPS (10 μg or 50 μg) on day 15.5 of pregnancy. Parturition was monitored from day 15.5 through day 21.5. Birth timing was documented upon delivery of the first pup or first observation of pups.

### Cell lines.

The human choriocarcinoma cell line BeWo (ATCC) was maintained at 37°C, 5% CO_2_, with DMEM/F12 (1:1) medium (Gibco) supplemented with 10% FBS (Life Technologies). For trophoblast syncytialization, BeWo cells seeded at 1.5 × 10^5^ cells/well in 24-well plates were cultured in medium with DMSO (vehicle) or 50 mM forskolin (Sigma-Aldrich) for 72 hours.

Primary human trophoblasts were isolated from cesarean-sectioned singleton term placentas without pregnancy complications by using the trypsin-DNase-dispase/Percoll method described previously (38). To purify primary CTBs, the trophoblasts were grown in MEM supplemented with 10% FBS for 4 hours and then washed 3 times with PBS to remove the syncytial fragments, which have lower affinity for the culture dish. After 24 hours, primary CTBs were transferred to phenol red–free DMEM with 10% charcoal-stripped FBS (Life Technologies) and continuously cultured for an additional 48 hours to induce differentiation into multinucleated STBs.

### Placental explants.

Mouse placentas were harvested on day 16.5 of gestation. The fetal compartments of the placentas were carefully dissected from connecting maternal tissues, including the myometrium and decidua. Each placenta was cut in half, rinsed thoroughly in cold PBS, and dissected into approximately 5-mm pieces. Mouse placental explants were seeded on collagen I–coated 12-well plates and cultured in DMEM with 10% FBS.

### siRNA transfection of STBs.

BeWo cells were cultured in medium with forskolin for 24 hours and then transfected with siRNA-targeting *ATG16L1* or control siRNA and Trans-X2 transfection reagent (Mirus). BeWo cells were grown in medium containing siRNA and forskolin for an additional 48 hours to form STBs.

### Colony formation assay.

Cell cultures were infected with a pathogenic *E*. *coli* clinical isolate, UTI89 (1 × 10^8^ CFU/ml), for 2 hours, treated with media containing gentamicin (50 μg/ml, Invitrogen) for 2 hours to kill extracellular bacteria, washed 3 times with PBS, and then lysed by the addition of 500 μl PBS with 0.1% Triton for 10 minutes. Cell lysates were transferred to 96-well plates, and serial dilutions of each lysate were added to LB plates and incubated at 37°C overnight, after which colonies were counted.

### Immunoﬂuorescence microscopy.

Cells cultured in Chamber Slides (Nunc) were fixed in 4% paraformaldehyde in PBS for 30 minutes at room temperature. Slides were stained for presence of bacteria and other markers as described previously (5). The following primary antibodies were used: rabbit polyclonal antibody to *E*. *coli* (1:500, United States Biological, E3500-26) and mouse monoclonal antibody to E-cadherin (1:500, BD, 610181). After 3 PBS washes at room temperature, antigen-antibody complexes were detected with species-specific Alexa Fluor 488 and 594–conjugated secondary antibodies (1:500, Invitrogen). Slides were stained with DAPI for 10 minutes and mounted with Prolong Gold (Life Technologies). Images were obtained with a Zeiss Apotome microscope using ×20 or ×60 oil immersion objectives.

### Hematoxylin and eosin staining and immunohistochemistry.

Human and mouse placenta samples were fixed with 4% paraformaldehyde in PBS for 24 hours and then embedded in paraffin. For histology, 5-mm sections were stained with Mayer’s Hematoxylin and Eosin Y solutions (Sigma-Aldrich). Immunohistochemistry was done as described previously (37). The following primary antibodies were used: anti-LC3 (1:200; Novus, NB600-1384), anti-P62 (1:500; Santa Cruz Biotechnology, SC28359), and anti-cytokeratin (1:1,000; DAKO, Z0622).

### TEM.

BeWo cells were prepared for TEM analysis as described previously (39). TEM sections were imaged using a JEOL 1200 EX transmission electron microscope (JEOL USA Inc.).

### Western blotting.

Total protein was extracted form frozen human and mouse placenta samples and homogenized in RIPA buffer (Cell Signaling Technology) with protease inhibitor cocktails (Sigma-Aldrich). Equivalent amounts of total protein, determined by BCA assays (Thermo Scientific), were separated on 4%–20% Mini-PROTEAN precast gels (Bio-Rad) and transferred to PVDF membranes. The following primary antibodies and dilutions were used: anti-LC3B (1:1,000; Novus, NB600-1384), anti-P62 (1:1,000; Abcam, ab56416), anti-Atg7 (1:1,000; Sigma-Aldrich, A2856), anti-Atg16L1 (1:1,000; Sigma-Aldrich, A7356), anti-Beclin1 (1:1,000; Cell Signaling, 3738s), and anti-Actin (1:2,000; Cell Signaling, 3700s). ImageJ (NIH) was used for densitometry of Western blots.

### Statistics.

Data are presented as mean ± SEM. The Mann-Whitney *U* test was performed on comparisons between two groups, and the Kruskal-Wallis test was performed with Dunnett’s post-test for multiple comparisons. Two-way ANOVA with a Bonferroni post-hoc test was used for multiple pairwise comparisons between different groups. Graphpad Prism 5.0 was used for all analyses. *P* < 0.05 was considered significant.

### Study approval.

This study was approved by the Washington University School of Medicine human studies review board (IRB ID 201012734). All animal procedures were reviewed and approved by the animal studies committee of the Washington University School of Medicine.

## Author contributions

BC and CM performed experiments. BC and IUM designed the research, analyzed data, and wrote the manuscript.

## Supplementary Material

Supplemental data

## References

[1] Robbins JR, Skrzypczynska KM, Zeldovich VB, Kapidzic M, Bakardjiev AI (2010). Placental syncytiotrophoblast constitutes a major barrier to vertical transmission of *Listeria monocytogenes*. PLoS Pathog.

[2] Zeldovich VB (2013). Placental syncytium forms a biophysical barrier against pathogen invasion. PLoS Pathog.

[3] Huppertz B (2008). The anatomy of the normal placenta. J Clin Pathol.

[4] Zeldovich VB, Robbins JR, Kapidzic M, Lauer P, Bakardjiev AI (2011). Invasive extravillous trophoblasts restrict intracellular growth and spread of *Listeria monocytogenes*. PLoS Pathog.

[5] Cao B, Mysorekar IU (2014). Intracellular bacteria in placental basal plate localize to extravillous trophoblasts. Placenta.

[6] Levine B, Mizushima N, Virgin HW (2011). Autophagy in immunity and inflammation. Nature.

[7] Deretic V, Saitoh T, Akira S (2013). Autophagy in infection, inflammation and immunity. Nat Rev Immunol.

[8] Bauckman KA, Owusu-Boaitey N, Mysorekar IU (2015). Selective autophagy: xenophagy. Methods.

[9] Oh SY, Choi SJ, Kim KH, Cho EY, Kim JH, Roh CR (2008). Autophagy-related proteins, LC3 and Beclin-1, in placentas from pregnancies complicated by preeclampsia. Reprod Sci.

[10] Hung TH, Chen SF, Lo LM, Li MJ, Yeh YL, Hsieh TT (2012). Increased autophagy in placentas of intrauterine growth-restricted pregnancies. PLoS One.

[11] Doulaveris G, Orfanelli T, Benn K, Zervoudakis I, Skupski D, Witkin SS (2013). A polymorphism in an autophagy-related gene, ATG16L1, influences time to delivery in women with an unfavorable cervix who require labor induction. J Perinat Med.

[12] Campbell MK, Challis JR, DaSilva O, Bocking AD (2005). A cohort study found that white blood cell count and endocrine markers predicted preterm birth in symptomatic women. J Clin Epidemiol.

[13] Klionsky DJ (2012). Guidelines for the use and interpretation of assays for monitoring autophagy. Autophagy.

[14] Hung TH, Hsieh TT, Chen SF, Li MJ, Yeh YL (2013). Autophagy in the human placenta throughout gestation. PLoS One.

[15] Drewlo S, Baczyk D, Dunk C, Kingdom J (2008). Fusion assays and models for the trophoblast. Methods Mol Biol.

[16] Cadwell K (2008). A key role for autophagy and the autophagy gene Atg16l1 in mouse and human intestinal Paneth cells. Nature.

[17] Elovitz MA, Mrinalini C (2004). Animal models of preterm birth. Trends Endocrinol Metab.

[18] Zeldovich VB, Bakardjiev AI (2012). Host defense and tolerance: unique challenges in the placenta. PLoS Pathog.

[19] Khan MY, Mah MW, Memish ZA (2001). Brucellosis in pregnant women. Clin Infect Dis.

[20] Carcopino X, Raoult D, Bretelle F, Boubli L, Stein A (2009). Q Fever during pregnancy: a cause of poor fetal and maternal outcome. Ann N Y Acad Sci.

[21] Lamont RF (2011). Listeriosis in human pregnancy: a systematic review. J Perinat Med.

[22] Cemma M, Brumell JH (2012). Interactions of pathogenic bacteria with autophagy systems. Curr Biol.

[23] Miner JJ (2016). Zika virus infection during pregnancy in mice causes placental damage and fetal demise. Cell.

[24] Mysorekar IU, Diamond MS (2016). Modeling Zika virus infection in pregnancy. N Engl J Med.

[25] Sapparapu G Neutralizing human antibodies prevent Zika virus replication and fetal disease in mice. Nature.

[26] Bildirici I, Longtine MS, Chen B, Nelson DM (2012). Survival by self-destruction: a role for autophagy in the placenta?. Placenta.

[27] Wang C (2012). Atg16L1 deficiency confers protection from uropathogenic *Escherichia coli* infection in vivo. Proc Natl Acad Sci U S A.

[28] Marchiando AM (2013). A deficiency in the autophagy gene Atg16L1 enhances resistance to enteric bacterial infection. Cell Host Microbe.

[29] Conway KL (2013). Atg16l1 is required for autophagy in intestinal epithelial cells and protection of mice from Salmonella infection. Gastroenterology.

[30] Maurer K, Reyes-Robles T, Alonzo F, Durbin J, Torres VJ, Cadwell K (2015). Autophagy mediates tolerance to Staphylococcus aureus alpha-toxin. Cell Host Microbe.

[31] Gawriluk TR, Ko C, Hong X, Christenson LK, Rucker EB (2014). Beclin-1 deficiency in the murine ovary results in the reduction of progesterone production to promote preterm labor. Proc Natl Acad Sci U S A.

[32] Hirota Y, Cha J, Yoshie M, Daikoku T, Dey SK (2011). Heightened uterine mammalian target of rapamycin complex 1 (mTORC1) signaling provokes preterm birth in mice. Proc Natl Acad Sci U S A.

[33] Agrawal V (2015). Altered autophagic flux enhances inflammatory responses during inflammation-induced preterm labor. Sci Rep.

[34] Broz P, Monack DM (2011). Molecular mechanisms of inflammasome activation during microbial infections. Immunol Rev.

[35] Symington JW (2015). ATG16L1 deficiency in macrophages drives clearance of uropathogenic E. coli in an IL-1β-dependent manner. Mucosal Immunol.

[36] Pontillo A, Girardelli M, Agostinis C, Masat E, Bulla R, Crovella S (2013). Bacterial LPS differently modulates inflammasome gene expression and IL-1β secretion in trophoblast cells, decidual stromal cells, and decidual endothelial cells. Reprod Sci.

[37] Stout MJ, Cao B, Landeau M, French J, Macones GA, Mysorekar IU (2015). Increased human leukocyte antigen-G expression at the maternal-fetal interface is associated with preterm birth. J Matern Fetal Neonatal Med.

[38] Kliman HJ, Nestler JE, Sermasi E, Sanger JM, Strauss JF (1986). Purification, characterization, and in vitro differentiation of cytotrophoblasts from human term placentae. Endocrinology.

[39] Bauckman KA, Mysorekar IU (2016). Ferritinophagy drives uropathogenic *Escherichia coli* persistence in bladder epithelial cells. Autophagy.

