# Supplementary material for: ATG16L1 governs placental infection risk and preterm birth in mice and women
Source: JCI Insight. 2016 Dec 22;1(21):e86654. doi: 10.1172/jci.insight.86654 (PMC5161251; doi:10.1172/jci.insight.86654)

Supplemental Table 1. Clinical characteristics of patients.

Characteristics	**Early preterm (n=10)**	**Late preterm (n=10)**	**Term (n=20)**
**Age**	28.10±1.99	30.10±2.47	30.90±1.24
**Gestational age**	27.55±2.10	34.48±2.17	39.01±1.19
**Maternal white blood cell count** ($\times 10^3$/µL)	17.54±9.12	10.20±3.00	10.08±2.24
**Maternal body temperature** (°F)	98.64±0.52	98.52± 0.92	98.11±0.60
**Chorioamnionitis**	4	4	0
**Usage of antibiotics**	9	7	12

Values are mean and standard deviation or number

Supplemental Figure Legends:

Supplemental Figure 1. **Correlation between mRNA levels of *ATG16L1* and *LC3* in human placentas.**

r = Pearson's correlation. **P* < 0.05

Supplemental Figure 2. Primary human syncytiotrophoblasts exhibit increased autophagy flux. Representative western blot detection of LC3 and P62 in human primary CTBs and STBs. Bar graphs represent mean values ± s.e.m. of n=3 independent experiments. **P* < 0.05 by Kruskal-Wallis test with Dunnetts's post-test.

Supplemental Figure 3. E. coli can localize to autophagosomes in trophoblasts. BeWo cells, pretreated with DMSO, Rapamycin and 3-MA, were infected with E.coli. Representative TEM images of BeWo cells collected at 2h pretreatment, 30 min and 2h post infection.

Supplemental Figure 4. **Normal reproductive phenotypes in $Atg16L1^{HM}$ breeders.**

A-B. Average weight of mouse placentas (A) and fetuses (B) of the indicated genotypes at D16.5. NS, not significant. C. Number of mouse placentas of the indicated genotypes. D. H&E staining and immunohistochemistry (IHC) of WT and HM placentas. CK, cytokeratin; De, decidua, Jz, junctional zone, La, labyrinth.

Supplemental Figure 5. **Atg16L1 deficiency decreases autophagic flux in mouse placenta**

Representative Western blot detection of LC3 and P62 in cells from placentas of the indicated genotypes.

Supplemental Figure 6. **Mouse placental explant model recapitulates normal morphology and trophoblast marker expression**

(A) H&E and (B) cytokeratin (green) and nuclear dye (blue) staining of mouse placental explants.

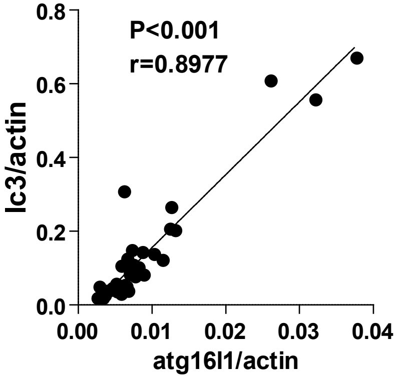
P<0.001
r=0.8977
lc3/actin
atg16l1/actin
0.0
0.2
0.4
0.6
0.8
0.00
0.01
0.02
0.03
0.04

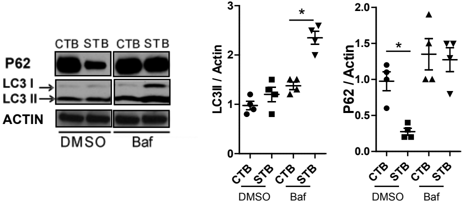

CTB STB CTB STB
P62
LC3 I
LC3 II
ACTIN
DMSO
Baf
LC3II / Actin
3
2
1
0
*
CTB
STB
CTB
STB
DMSO
Baf
P62 / Actin
2.0
1.5
1.0
0.5
0.0
*
CTB
STB
CTB
STB
DMSO
Baf

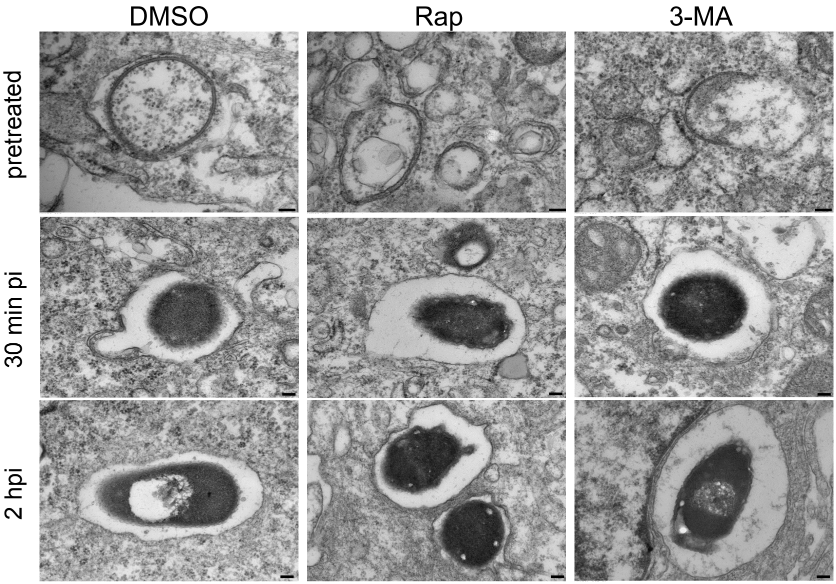

DMSO
Rap
3-MA
pretreated
30 min pi
2 hpi

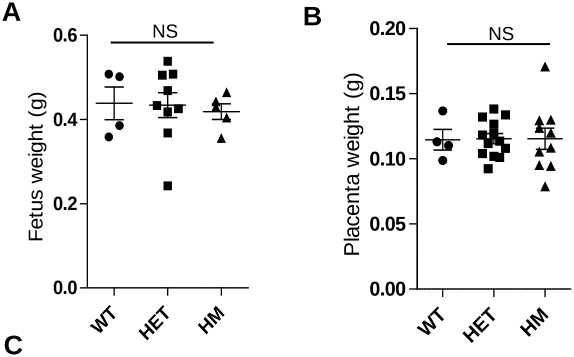

C

Gentoype of mouse placentas

	WT	HET	HM
Number	22	50	25
Percentage (%)	22.7	51.5	25.8

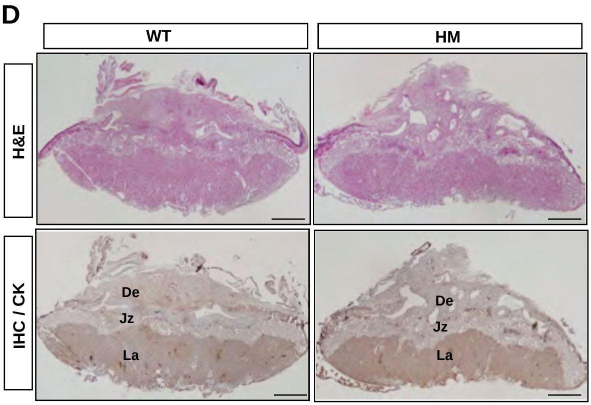

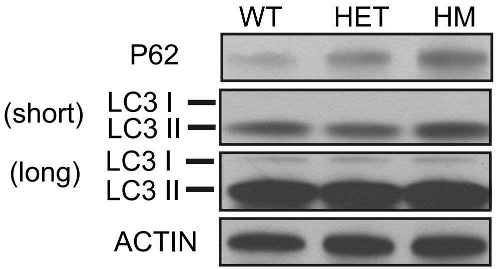
WT
HET
HM
P62
(short)
LC3 I
LC3 II
(long)
LC3 I
LC3 II
ACTIN

A B

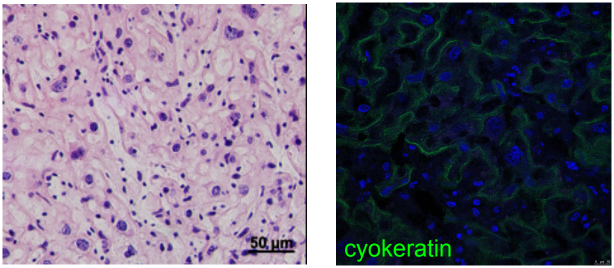

Supplement: Supplemental data [file jciinsight-1-86654-s001.pdf]
